# The Involvement of the *Cas9* Gene in Virulence of *Campylobacter jejuni*

**DOI:** 10.3389/fcimb.2018.00285

**Published:** 2018-08-20

**Authors:** Muhammad A. B. Shabbir, Yanping Tang, Zihui Xu, Mingyue Lin, Guyue Cheng, Menghong Dai, Xu Wang, Zhengli Liu, Zonghui Yuan, Haihong Hao

**Affiliations:** ^1^China MOA Laboratory for Risk Assessment of Quality and Safety of Livestock and Poultry Products, Huazhong Agricultural University, Wuhan, China; ^2^National Reference Laboratory of Veterinary Drug Residues, Huazhong Agricultural University, Wuhan, China

**Keywords:** *Campylobacter jejuni*, *cas9*, virulence, transcriptome analysis, differential gene expression

## Abstract

*Campylobacter jejuni* is considered as the leading cause of gastroenteritis all over the world. This bacterium has the CRISPR–*cas9* system, which is used as a gene editing technique in different organisms. However, its role in bacterial virulence has just been discovered; that discovery, however, is just the tip of the iceberg. The purpose of this study is to find out the relationship between *cas9* and virulence both phenotypically and genotypically in *C*. *jejuni* NCTC11168. Understanding both aspects of this relationship allows for a much deeper understanding of the mechanism of bacterial pathogenesis. The present study determined virulence in wild and mutant strains by observing biofilm formation, motility, adhesion and invasion, intracellular survivability, and cytotoxin production, followed by the transcriptomic analysis of both strains. The comparative gene expression profile of wild and mutant strains was determined on the basis of De-Seq transcriptomic analysis, which showed that the *cas9* gene is involved in enhancing virulence. Differential gene expression analysis revealed that multiple pathways were involved in virulence, regulated by the CRISPR-*cas9* system. Our findings help in understanding the potential role of *cas9* in regulating the other virulence associated genes in *C. jejuni* NCTC11168. The findings of this study provide critical information about *cas9*'s potential involvement in enhancing the virulence of *C. jejuni*, which is a major public health threat.

## Introduction

Campylobacteriosis is one of the most common intestinal diseases (WHO Advisory Group on Integrated Surveillance of Antimicrobial Resistance (AGISAR)., 2011), mainly caused by *C. jejuni* in humans (Tauxe, 2001). This bacteria infects humans by contaminating poultry products, milk and water (Manavathu et al., 1988; Chai et al., 2009; Pitkänen, 2013; Huang et al., 2015). Outbreaks of *C. jejuni* infection in humans are sporadic and relatively rare. However, serious possible consequences of *Campylobacter* infection include an autoimmune mediated disease known as Guillain-Barre Syndrome (GBS) and the closely related Miller Fisher Syndrome (Han et al., 2007). The pathogenesis of *C*. *jejuni* is achieved by numerous factors interacting together, including motility, adhesion, invasion, and toxin production (Bolton, 2015).

Motility is the key for bacteria to against various chemotactic conditions encountered in the gastrointestinal tract (Bolton, 2015). In the presence of viscous substances, *Campylobacter* shows unusual motility (Ferrero and Lee, 1988). *C. jejuni* motility factors include *flaA* (Lertsethtakarn et al., 2011), *flab*, and *flgI* (Sommerlad and Hendrixson, 2007), *fliA* and *rpoN* (Hendrixson, 2006). *C. jejuni* adherence to the host intestinal epithelial cells is also important for colonization, which is facilitated by various adhesions present on bacterial surfaces (Jin et al., 2001). These adhesion factors are *cadF* (Konkel et al., 1997), *capA*, and *jlpA* (Jin et al., 2001), and *virB11* (Dasti et al., 2010).

Along with motility, flagella also function as an export apparatus (T3SS) during the host invasion (Poly and Guerry, 2008). In various studies, it was revealed that the flagellar apparatus functions as a type III secretion system (T3SS), transporting *C. jejuni* invasion antigens (*Cia*) into the host cell (Ziprin et al., 2001; Konkel et al., 2004; Biswas et al., 2007). The invasion antigen (*CiaI*) is secreted by *C. jejuni* via T3SS and consists of amino-terminal type III secretion sequences (Konkel et al., 2004). It enables the *C. jejuni* to survive within epithelial cells (Buelow et al., 2011). *Campylobacter* invasion factors include *flaC, flhA, flhB, fliO, flip*, and *fliR* (Carrillo et al., 2004), *ciaB* (Konkel et al., 2004), *ciaC* (Christensen et al., 2009), *ciaI* (Buelow et al., 2011), *iamA* (Carvalho et al., 2001), *htrA* (Bæk et al., 2011) and *virK* (Poly et al., 2007). *C. jejuni* produces a number of cytotoxins (McFarland and Neill, 1992); however, detailed study has been conducted only on cytolethal distending toxin (CDT; Pickett and Whitehouse, 1999). Nucleotide sequence analysis revealed that the cluster of CDT genes consists of *cdtA, cdtB*, and *cdtC* in *C. jejuni* (Pickett et al., 1996; Purdy et al., 2000; Lara-Tejero and Galán, 2001; Dasti et al., 2010). Along with CDT, *C. jejuni* also have *cgtB* and *wlaN* toxin factors (Gilbert et al., 2000; Linton et al., 2000).

Clustered regularly interspaced short palindromic repeats (CRISPRs) are short sequences of repeats that have been found in different bacterial genomes (Price et al., 2007). The term CRISPR was first used in 2002, and refers to the specific structure of loci. The CRISPR system is divided into three types on the basis of specific proteins present in each system. CRISPR system type I has *cas3*, type II has *cas9*, and type III has *cas10* protein. The CRISPR type II system present in *C. jejuni* and *cas9* plays an important role in the attachment and invasion of these bacteria in the colorectal epithelial cell model (Shabbir et al., 2016). Adherence to the host epithelial cells play an important role in bacterial infection (Soto and Hultgren, 1999). In 2013, scientists observed that *cas9* of *Francisella novicida* uses CRISPR cas-associated RNA (*scaRNA*) for the repression of an endogenous transcription, which encodes a bacterial lipoprotein. This helps in the intracellular survivability of the pathogen within the host, as TLR2 activation is dampened (Sampson et al., 2014). Scientists in 2013 also reported that *C. jejuni* (*cst-II*-positive) strains almost lost their virulence after the deletion of the *cas9* gene, and they also observed that GBS patients' serum was highly visible in the human immune system (Louwen et al., 2013).

The mechanism underlying the role of *cas9* as a virulence factor in *C. jejuni* is not completely understood. Therefore, the purpose of the current study was to observe how the *cas9* gene enhances virulence by regulating the virulence of associated genes. Therefore, transcriptomic analysis was performed after the deletion of the *cas9* gene in *C. jejuni* and the wild type was compared with Δ*cas9* mutant strain to find out the role of *cas9* as a virulence factor in *C. jejuni*. Along with transcriptomic profiling, we also checked and compared the virulence of *C. jejuni* wild and Δ*cas9* mutant strains by phenotypic methods, such as determination of cytotoxin assay, biofilm assay, adhesion, invasion, and intra-cellular survivability assay, along with motility. Our results showed that the *cas9* gene plays a critical role in enhancing virulence. The findings of the present study shed light on the importance of the *cas9* gene in the virulence of *C. jejuni*, which is a major public health concern.

## Materials and methods

### Bacterial strain and growth condition

The *C. jejuni* NCTC11168 strain used in this study was provided by China's center for disease control and prevention. *C. jejuni* was grown on Skirrow agar supplemented with 5% sheep blood and Skirrow addition agent at 42°C under microaerobic conditions (5% O_2_, 10% CO_2_, and 85% N_2_) for 24–48 hours (h).

### Construction of *Cas9* isogenic mutant

The *cas9* (Gene ID: 905809) gene was found by an NCBI blast in the genome of *C. jejuni* NCTC11168. For the amplification of the *cas9* gene, adjacent upstream flanking sequences (about 1300bp) and downstream flanking sequences (about 1300bp) were used. The kanamycin resistance (*Kan*) cassette (pmdT- TKarmA- TKarmB -Kan- ef1a- BFP-WPRE-polyA) was amplified by PCR, which was donated by Huazhong agricultural university. These three fragments were ligated together by adopting the method of overlapping PCR. This overlapping PCR product was then ligated to the pGem-Teasy vector (Promega; Hao et al., 2016). The resulting constructs were sequenced and electroporated into the *C. jejuni* NCTC11168 strain and recombinants were selected on blood agar plates containing 5% sheep blood (Becton Dickinson) and 50 μg/mL kanamycin (Sigma-Aldrich). Junction PCR was used to confirm the double crossover events and the orientation of the *Kan* resistance cassette was assessed, with primers up and downstream of the area involved in the homologous recombination and primers (see S1 Table).

### Electro-transformation of the *C. jejuni* isolate

For the electro-transformation of *C. jejuni*, 5% sheep blood agar plates (with and without antibiotic) were put into a 37°C incubator, to pre-warm. *C. jejuni* was harvested from the inoculated Skirrow blood agar plate (Becton Dickinson) using Luria broth (LB; Becton Dickinson) supplemented with 1 × nonessential amino acids (NEAA; Invitrogen). *C. jejuni* was pelleted by centrifugation for 5 min at 8,000 rpm and re-suspended with 1.5 mL of LB containing 1 × NEAA (Invitrogen), which was repeated one time. For resuspension, a transformation buffer (0.5 mL) without 1 × NEAA (Invitrogen) was used and aliquoted in 1.5 mL centrifuge tubes having 100 μL samples of the competent cells. To make Δ*cas9* mutants, plasmid DNA (2 μg) was transformed into the 100 μL sample of competent cells. The mixture was transferred to an electroporation cuvette and pulsed at 2.5 kV, 200 Ω, and 25 μF. Then 1 mL of LB supplemented with 1 × NEAA (Invitrogen) was added to the cuvette and re-suspended by pipetting thoroughly. This mixture (200 μL) from the cuvette was plated and spread onto the surface of pre-warmed Columbian blood agar plates (Becton Dickinson). Plates were incubated for 5 h at 42°C under micro-aerophilic conditions. The cells were recovered from these plates using 2 mL of LB supplemented with 1 × NEAA (Invitrogen). After centrifugation, suspension was spread on the surface of a new pre-warmed blood agar plate containing 50 μg/mL kanamycin (Sigma-Aldrich) for the selection of knock-out mutants, and ampicillin (Sigma-Aldrich) was used with a concentration of 50 μg/mL to screen for supplemented Δ*cas9* clones. Plates were incubated at 42°C under micro-aerophilic conditions for 2–5 days, until colonies were visible. Colonies resistant to ampicillin or kanamycin were passaged five times on new plates to generate stable clones. From these stable clones, a PCR assay was used to test for the correctness of the supplementation assay. For supplementation, we used the primer pairs presented in S2 Table to confirm that the supplemented strains had the gene inserted in the same sense orientation (Louwen et al., 2013).

### Construction of complemented mutant strain

Complemented *cas9* mutant strain was constructed according to the previously described method (Howard et al., 2009). Briefly, target gene Cj1523c (*cas9*, Gene ID: 905809) was amplified from extracted genomic DNA of *C. jejuni* NCTC11168. The amplified gene product was subsequently cloned into the pGEM-Teasy vector. Sequencing was performed to confirm the absence of mutation in the target gene. The target gene was electroporated into the *cas9* mutant strain and transformants were selected on skirrow's agar plates. Positive colonies were confirmed by PCR using the targeted gene primers (S2 Table).

### Determination of standard growth curve

The standard growth curves of *C. jejuni* NCTC11168, complemented mutant and Δ*cas9* mutant strains were determined as per method described previously (Davis and DiRita, 2008). Biphasic MH (50% MH broth and MH agar) medium was used to perform growth curves. The inoculum of three strains (1 × 10^6^ CFU/mL) was incubated separately under micro-aerobic conditions for 16 to 18 h. Cultures of wild, complemented and mutant strains were suspended to an optical density of 0.4 at 600 nm. Suspensions were diluted (1:10) and inoculated (80 μL) into 20 mL of biphasic medium. Biphasic medium of each strain was incubated under both static and shaking micro-aerobic conditions. Orbital shaker was used to determine the growth curves under shaking condition according to the previously described method (Ghaffar et al., 2015). Viable colonies of three strains were determined at 0 h to 40 h with 4 h interval.

### Biofilm assays

To measure the biofilm formation of the *C. jejuni* and its Δ*cas9* mutant strain, a microtiter plate biofilm test was used as previously described for *C. jejuni* and other bacteria (Stepanović et al., 2000) with some modifications. Briefly, 200 uL of *C. jejuni* and Δ*cas9* mutant strain fresh culture (OD_570_ = 0.05) were added to 96-well microtiter plates (flat bottom) and incubated at 42°C under microaerobic conditions for 24, 48, and 72 h. After every mentioned time interval, wells were washed three times with PBS (phosphate buffered saline) to remove the planktonic cells. Methanol (200 uL) was added in all wells and incubated for 15 min, followed by drying at room temperature and subsequently, 200 uL of 0.1% Hucker crystal violet solution were added into the plates and incubated for 5 min at room temperature. PBS was used to wash out the unbound crystal violet, and the plates were dried at 60°C. Glacial acetic acid (30%) was used to dissolve the bounded crystal violet. A plate reader(BioTek, uQuant microplate reader, USA)was used to determine absorbance (570 nm). Sterile media in wells were used as a blank control. To report biofilm production in *C. jejuni* and Δ*cas9* mutant strains, their blank corrected absorbance values were used. Based on bacterial films (ODs), both strains were categorized into non-adherent (0), weakly (^*^), moderately (^**^), and strongly (^***^) adherent. For the microtitration test, three standard deviations of negative control were considered as the cut-off OD.

Assays were repeated three times with three technical replicates and the results were averaged.

### Adhesion and invasion assay

The adhesion and invasion of *C. jejuni* and Δ*cas9* mutant strains were determined as previously described for *C. jejuni* and other bacteria (Hao et al., 2016). Briefly, 5.0 × 10^7^ CFU/mL of the *C. jejuni* and Δ*cas9* mutant was used to inoculate the monolayers of macrophages RAW264.7 at multiplicity of infection (MOI) of 100. For adhesion and invasion to occur, the infected monolayers were incubated for 3 h. In order to determine the total number of adherent and internalized bacteria, Dulbecco's Modified Eagle's Medium (DMEM) without antibiotic was used for the washing (three times) of monolayers to get rid of the extracellular unbound bacteria. The intracellular bacteria were released by the lysis of monolayers. Invading bacteria were determined by washing the monolayers with aspirated DMEM medium and 100 ug/mL gentamicin was added for 2 h to kill the bound as well as extracellular bacteria. The intracellular bacteria were released after washing and lysis of monolayers. The adherent bacteria number was calculated by subtracting the number of internalized bacteria from the total number of adherent and internalized bacteria (Iqbal et al., 2016).

### Intracellular survivability assay

To test the intracellular survivability of *C. jejuni* and Δ*cas9* mutant strains within the macrophages RAW264.7, the post infection invasion period of both strains was extended to 3, 6, 12, 18, 24, 48, and 72 h. Same procedure was applied for washing and lysis of cells as described in the adhesion and invasion assay section. The intracellularly survived bacteria were counted by serial dilutions on Skirrow blood agar (Iqbal et al., 2016).

### Cytotoxin assay

RAW264.7 macrophages were used to determine the cytotoxic effects of the *C. jejuni* NCTC 11168 and Δ*cas9* mutant strains. *C*. *jejuni* can produce a unique cytotoxin (Guerrant et al., 1987). A cytotoxin assay was performed according to the previously described method (Guerrant et al., 2001). Briefly, *C. jejuni* and Δ*cas9* mutant strains were treated by 2000 μg/mL polymyxin B and incubated at 37°C for 1 h (Ashkenazi and Cleary, 1990); subsequently, centrifuged at 8,000 rpm for 20 min. The supernatant was filtered with a 0.22 μm filter. 0.3% TritonX-100-PBS was used as a positive control, and polymyxin B-treated microbial and polymyxin B-PBS were used as negative control. Suspensions of macrophages RAW264.7 (100 μL:5 × 105 cells) were placed into the microtitration plates for 1 to 3 h to allow adherence. The prepared serial twofold dilutions of *C. jejuni* and Δ*cas9* mutant strain filtrates were used for tissue culture monolayers in 100 μL volumes, and incubated at 37°C in 5% CO2 for 24 h. The monolayers were examined for the percentage of cells of round cells by phase-contrast microscopy. A Trypan Blue Dye uptake (A540 value) was used to determine the cell death and after that a staining technique known as “Giemsa staining” was used to correlate with normal cell morphology. The index of cytotoxin effect was calculated by the formula (Hao et al., 2016) 100 × {1–[A540 (test) A540 (positive)] ÷[A540(negative)–A540 (positive)]}.

### Motility test

The two strains (*C. jejuni* NCTC11168 and Δ*cas9* mutant strains) used to test mobility, a fresh culture of *C. jejuni* and Δ*cas9* mutant strains, were inoculated into Mueller Hinton (MH) broth and cultured to their logarithmic growth phase. Then 3 uL of bacteria were stabbed into 0.4 % MH agar and plates were incubated at 42°C under microaerobic conditions for 24 h. From each plate, motility was scored by measuring growth diameter (mm). Assays were repeated at least three times with three technical replicates and the results were averaged (Kim et al., 2015).

### Frozen transmission electron microscopy

The flagellar length of *C. jejuni* NCTC11168 and Δ*cas9* mutant strains were examined using frozen transmission electron microscopy according to a previously described method (Hao et al., 2017). Briefly, plates were washed with 2 mL PBS to get bacterial suspensions and spotted on carbon-coated copper grids. Bacterial cells were stained with 2% phosphotungstic acid (pH 6.7) for 1 min. Samples were observed employing a transmission electron microscope (H-7650, Japan).

### Transcriptome analysis by RNA-Seq

After the phenotypic determination of *C. jejuni* NCTC 11168 and Δ*cas9* mutant strains, RNA-Seq was performed as described previously (Iqbal et al., 2016). *C. jejuni* and Δ*cas9* mutant strains (two each sample) were harvested at log phase. By following the manufacturer's instructions, total RNAs were isolated from the samples using TRIzol (Invitrogen Inc., California, USA). RNase free DNase I (Ambion Inc., Texas, USA) was used to remove DNA and the quality of RNA was checked with the help of Agilent 2100 system with RIN (RNA integrity number) over 7. From total RNA, rRNA was removed by using the Ribozero kit and strand specific RNA-Seq protocol was followed on the illumine DE-Seq platform (Paired end sequencing; 100 bp fragment) at Shanghai personal biotechnology. Briefly, synthesis of cDNA first strand was carried out using the Super-ScriptII (Invitrogen, Carlsbad, CA) in the presence of random hexamer primers and synthesis of cDNA second strand was performed before end repair and dA tailing. Ligation of the DNA fragment was performed by using a truSeq adapter and amplified with truSeq PCR primers for sequencing. Reads more than 35 nucleotide (nt) were selected. Furthermore, paired reads were removed that were matched to the sliva database (http://www.arb-silva.de/download/arb-files/). With the help of the DE-Seq package, all genes' expression in different samples were transformed to count per gene (CPG) by using blind and fit-only parameters. From the respective repeats of *C. jejuni*, NCTC 11168 and Δ*cas9* mutant mean CPG gene expression were determined and comparison was made between the differentially expressed genes (DEGs) of both strains. The DEGs having *P*-value ≤ 0.05 with fold change ≥1.5 were regarded as up-regulated genes, while DEGs having fold change ≤ 1.5 were considered as down-regulated genes. For the functional gene classification, gene ontology (GO) from an internationally established system was followed. This system not only comprehensively describes properties of genes but also provides their products in any organism. Further analyses of DEGs were performed on the basis of biological process, molecular function and cellular components. The Kyoto Encyclopedia of genes and genomes (KEGG) database was used to find out the role of DEGs in various pathways. The data has been submitted to Gene expression omnibus (GEO) under the accession number SUB2870584.

### Validation by RT-qPCR

RNA sequencing results were verified by RT-qPCR. For that, seven genes were selected on the basis of their role in virulence and differential expression in RNA-Seq results. These included *Cj0762c* (aspartate aminotransferase), *Cj1523c* (CRISPR-associated protein), *Cj1222c* (two-component sensor histidine kinase), Cj0996 (GTP cyclohydrolase II), Cj0725c (molybdenum cofactor biosynthesis), *Cj0454c* (membrane protein), and *Cj0330c* (50S ribosomal protein L32). For RT-qPCR, a 16SrRNA gene was used as a housekeeping gene. Primers used in RT-qPCR are given in the S3 Table.

### Statistical analysis

For statistical analyses, SPSS version 22.0 (IBM corp., Armonk, NY, USA) was used to determine the mean ± standard deviation (MSD) and significance level by applying a two-tailed *t*-test comparing *C. jejuni* NCTC11168, and its Δ*cas9* mutant strain for *in vitro* experiments, such as biofilm formation, adhesion, and invasion, and intracellular survivability. A Pearson's correlation coefficient (r) was computed between RNA-Seq and RT-qPCR results with a significance cutoff of *P* ≤ 0.05.

## Results

### *Cas9* gene role on growth

**Figure 1:**
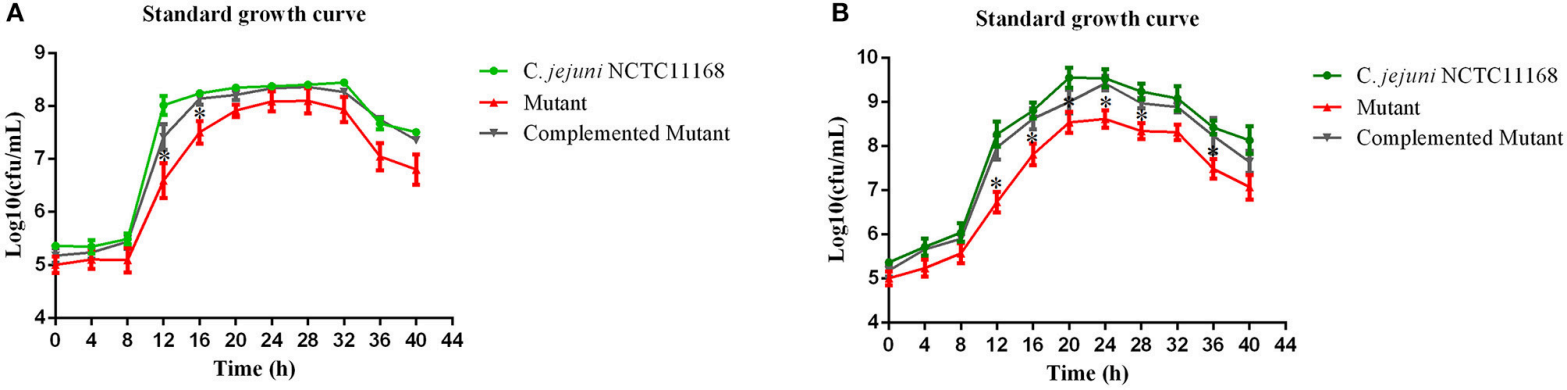
**(A,B)** Standard growth curve of mutant with its parent strain under static and shaking conditions. **(A)** represents the bacterial growth curve under static conditions, while **(B)** shows the bacterial growth under shaking conditions. The Y axis shows the growth of *C. jejuni* NCTC11168, complemented and Δ*cas9* mutant strains in Log10 (cfu/mL) in static condition. One asterisk (*) represent statistically significant differences at *P* ≤ 0.05, in comparisons of *C. jejuni* NCTC11168, complemented and the Δ*cas9* mutant.

*Campylobacter jejuni* NCTC11168, complemented mutant and Δ*cas9* mutant strains were grown separately in biphasic media of MH for the determination of standard growth curve. This experiment was performed to determine growth characteristics of three strains. Compared to growth of *C. jejuni* NCTC11168, complemented and Δ*cas9* mutant under static condition (Figure 1A), we found significant difference in growth between three strains, being grown under shaking conditions (Figure 1B).

### Biofilm formation of the *C. jejuni* NCTC11168 and mutant

**Figure 2:**
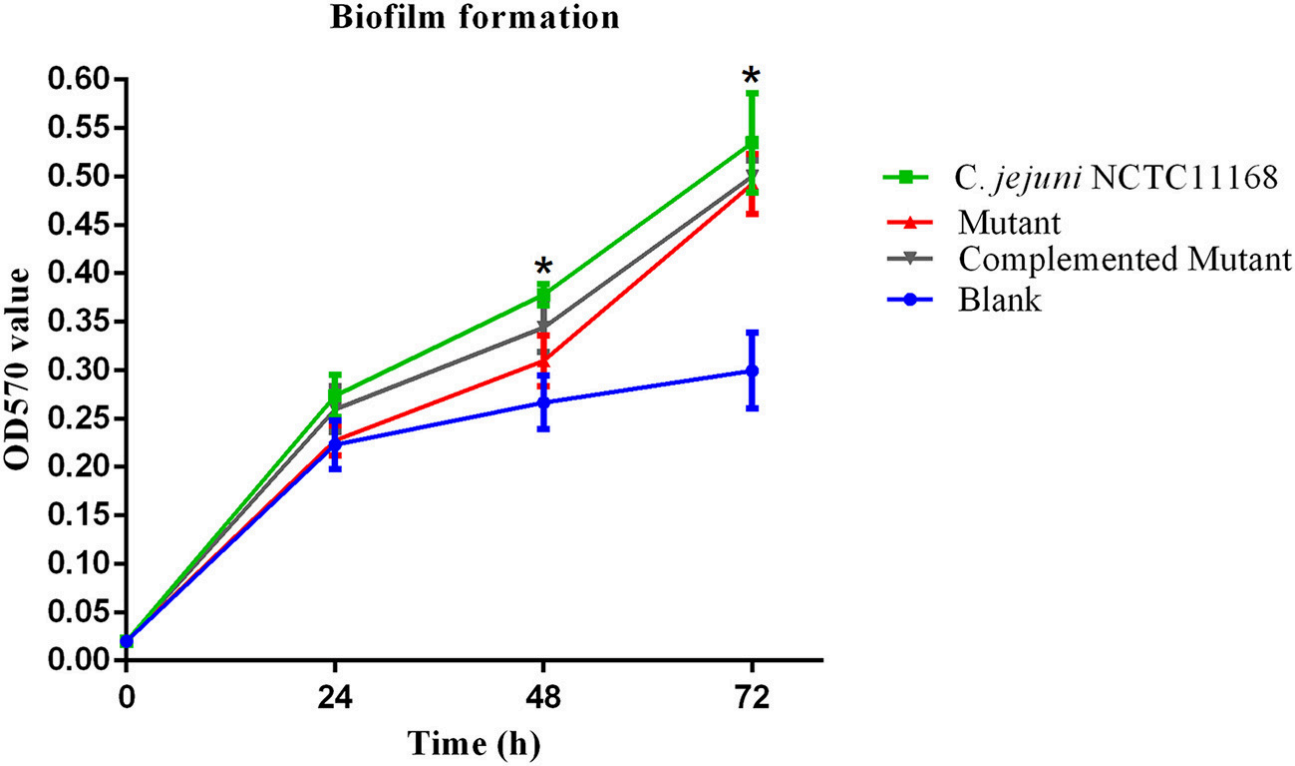
Biofilm formation of *C. jejuni* NCTC11168 and Δ*cas9* mutant at different time points. The Y axis is the OD_570_ value of crystal violet in biofilm. The results were obtained from three independent repeats. The asterisk (*) indicates statistical significance at *P* ≤ 0.05 for the comparison of *C. jejuni* NCTC11168, complemented, and the Δ*cas9* mutant.

It has been suggested that biofilm formation helps the *C. jejuni* to survive in the environment against various stressors. In other words, biofilm formation aids the virulence of bacteria. Therefore, we determined the biofilm of *C. jejuni* NCTC11168, complemented and Δ*cas9* mutant strains *in vitro* at different time intervals. The highest level of biofilm formation occurred after 72 h of incubation. It seemed that the level of biofilm formation had a positive correlation with the incubation time. Compared to the Δ*cas9* mutant strain, a significant difference in biofilm formation was observed in the *C. jejuni* NCTC11168 strain after each incubation time, indicating that the *C. jejuni* NCTC11168 strain may have stronger ability to form biofilm (Figure 2).

### Adhesion and invasion of *C. jejuni* NCTC11168 and mutant in macrophages RAW264.7

**Figure 3:**
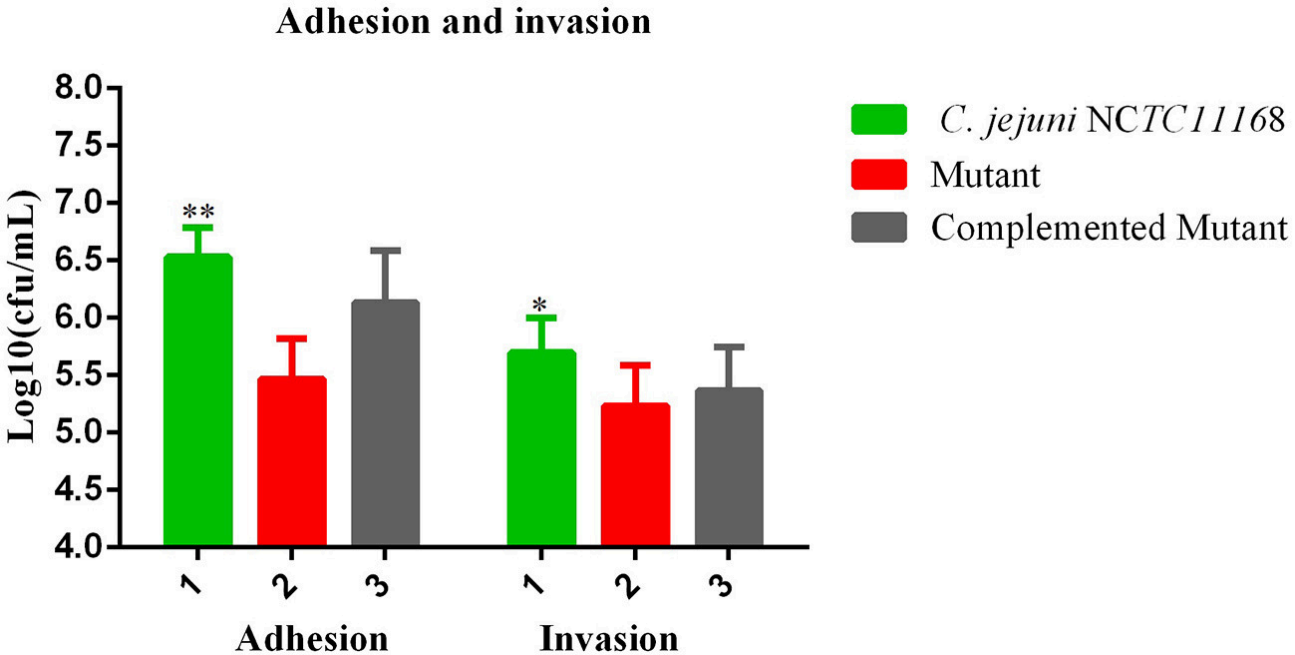
Adhesion and invasion of *C. jejuni* NCTC11168 and Δ*cas9* mutant on macrophage RAW 264.7 cells. The Y axis is the mean of log10 CFU/mL of each strain in the RAW264.7 cell. The results were obtained from three independent repeats. One asterisk (*) and two asterisks (**) represent statistically significant differences at *P* ≤ 0.05and *P* ≤ 0.01, respectively, in comparisons of *C. jejuni* NCTC11168, complemented, and the Δ*cas9* mutant.

Most of the bacteria having a CRISPR-cas type II system are considered pathogenic bacteria. Therefore, adhesion and invasion assay was performed to determine the role of the *cas9* gene in virulence. We observed that *C. jejuni* NCTC11168 exhibited remarkably higher adhesion and invasion of macrophage RAW264.7 cells than the Δ*cas9* mutant strain (Figure 3). A significant change of adhesion to macrophage cells (*P* < 0.05) was observed in *C. jejuni* NCTC1168 compared to its Δ*cas9* mutant strain. While, almost similar trend of complemented strain with *C. jejuni* NCTC11168 in terms of adhesion and invasion was observed.

### Intracellular survivability of *C. jejuni* NCTC11168 in macrophages RAW264.7

**Figure 4:**
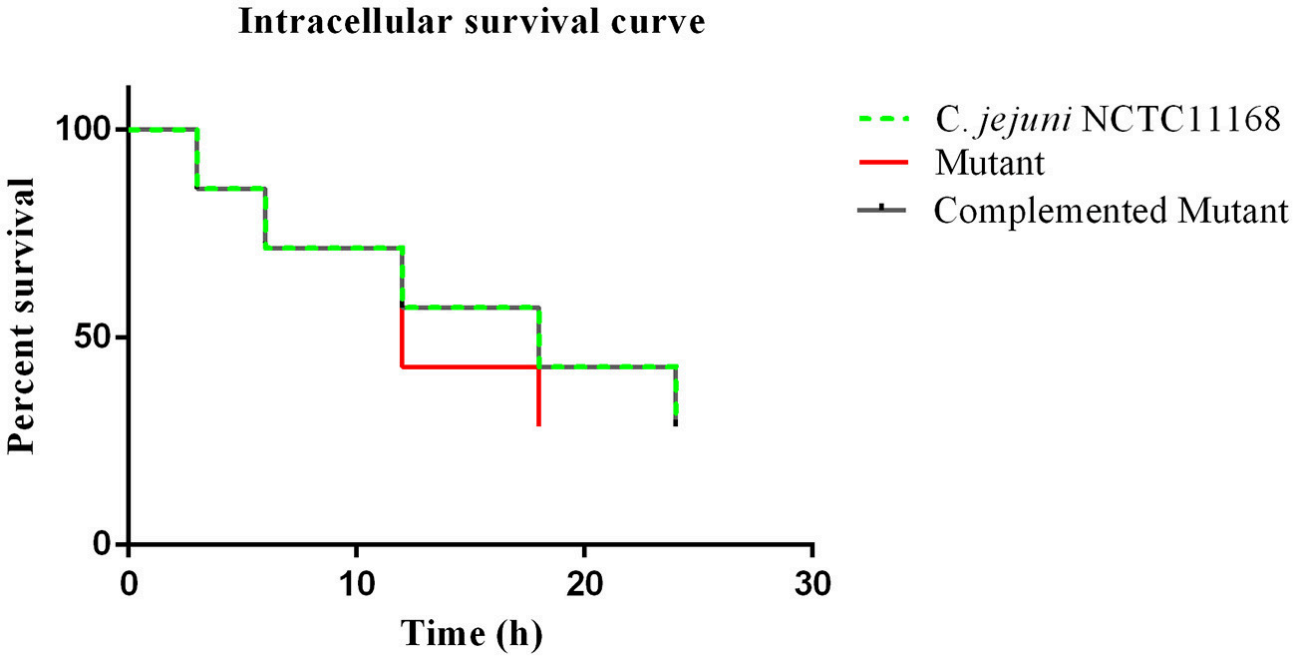
Intracellular survivability of *C. jejuni* NCTC11168 and Δ*cas9* mutant in macrophage RAW 264.7 cells. The Y axis shows the percent survival of *C. jejuni* NCTC11168, complemented and Δ*cas9* mutant strain in the RAW264.7 cell. The analysis was performed by using the Kaplan-Meier estimate method. The results showed that *C. jejuni* NCTC11168 and complemented strains had more survivability than Δ*cas9* mutant strain.

An intracellular survivability test was performed to determine whether the *cas9* gene has a role in the survivability of *C. jejuni* intracellularly or not. It was observed in the present study that the intracellular survivability of *C. jejuni* NCTC11168 and complemented strains are greater than that of the Δ*cas9* mutant strain (Figure 4). The Δ*cas9* mutant strain had a shorter survival time (18 h) than the *C. jejuni* NCTC11168 strain, which was able to survive for 24 h. However, a considerable decrease in the number of internalized bacteria was observed at all stages. At each time point, the post-infection number of surviving *C. jejuni* NCTC11168 was higher than the Δ*cas9* mutant, suggesting that the *C. jejuni* NCTC11168 strain had greater survivability.

### Cytotoxin of the *C. jejuni* NCTC11168 and Δ*cas9* mutant in macrophage cells

**Figure 5:**
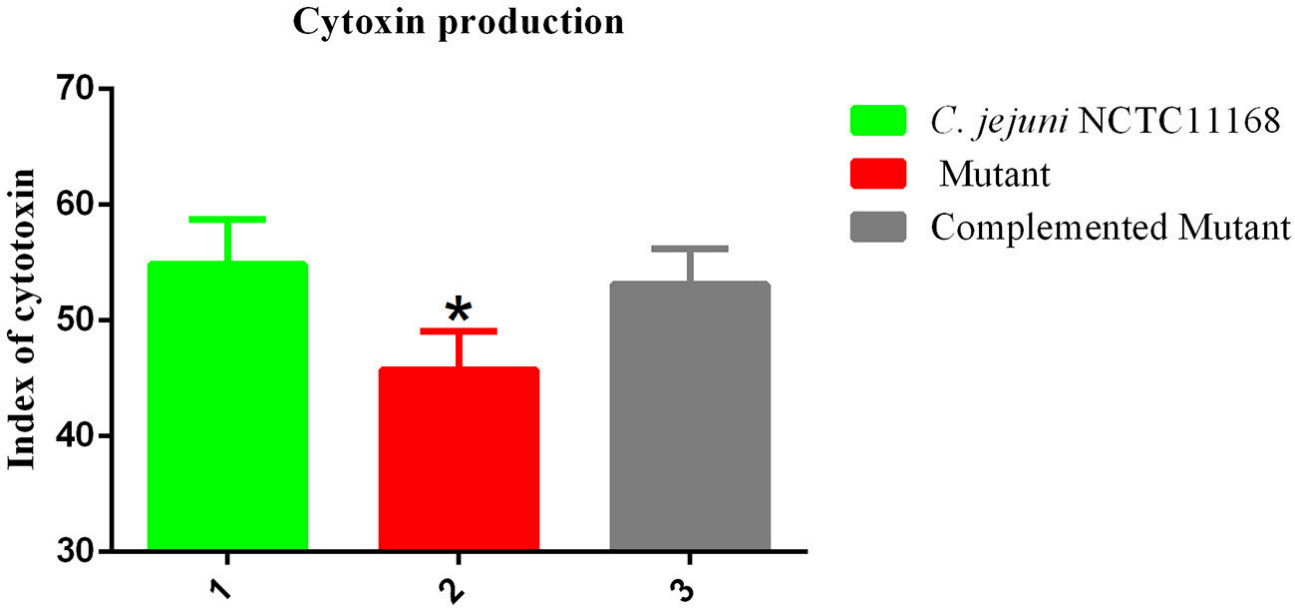
Cytotoxic production of the *C. jejuni* NCTC11168 and Δ*cas9* mutant in murine macrophage RAW264.7 cells. The Y axis is the index of cytotoxic production of each strain. The results were obtained from three independent repeats. One asterisk (*) indicates a statistically significant difference at *P* ≤ 0.05 between *C. jejuni* NCTC11168, complemented, and the Δ*cas9* mutant.

*Campylobacter jejuni* NCTC11168 produces toxins, which is another factor in virulence. Therefore, the cytotoxin production ability of *C. jejuni* NCTC11168, complemented and Δ*cas9* mutant strain was determined. The present study showed the killing effect (index) of cytotoxin released by *C. jejuni* NCTC11168, complemented and Δ*cas9* mutant strains in murine macrophage RAW264.7 cells (Figure 5). It was observed that *C. jejuni* NCTC11168 and complemented strains had the ability to produce more toxin than the Δ*cas9* mutant strain.

### Motility of the *C. jejuni* NCTC11168 and Δ*cas9* mutant

**Figure 6:**
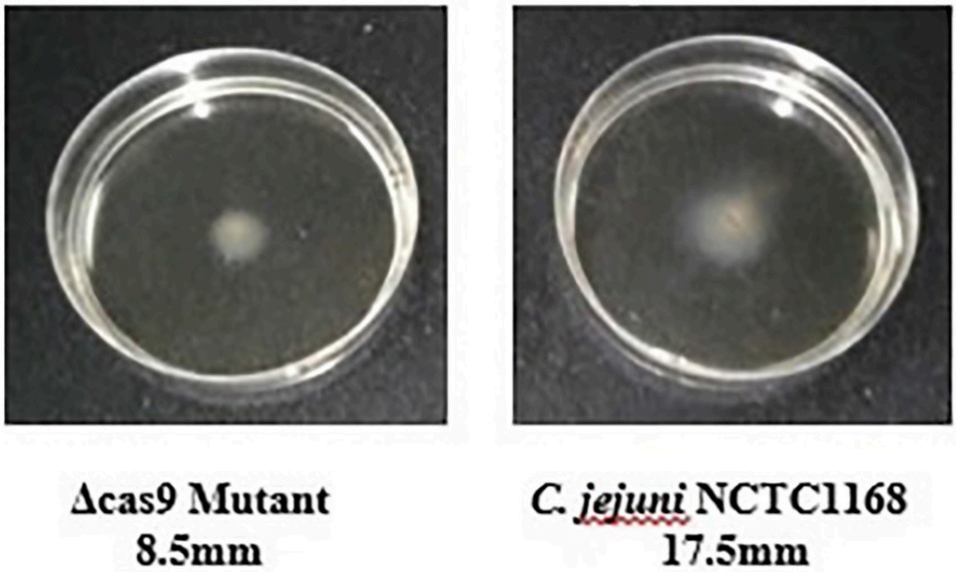
Motility of the *C. jejuni* NCTC 11168 and Δ*cas9* mutant strains on 0.4% MH agar plates. Motility is positively correlated to the diameter of the growth ring of each strain.

Flagella were also considered a virulence factor that helps in the motility and colonization of *C. jejuni* in the gastrointestinal tract of host. Therefore, motility of *C. jejuni* NCTC11168 and Δ*cas9* mutant strains were determined. The present study showed that there was an apparent difference between diameter growth rings of the *C. jejuni* NCTC11168 and Δ*cas9* mutant strains on a 0.4% MH agar plate (Figure 6). It was observed that Δ*cas9* mutant strains have less motility than the *C. jejuni* NCTC11168 strain.

### Frozen transmission electron microscopy

**Figure 7:**
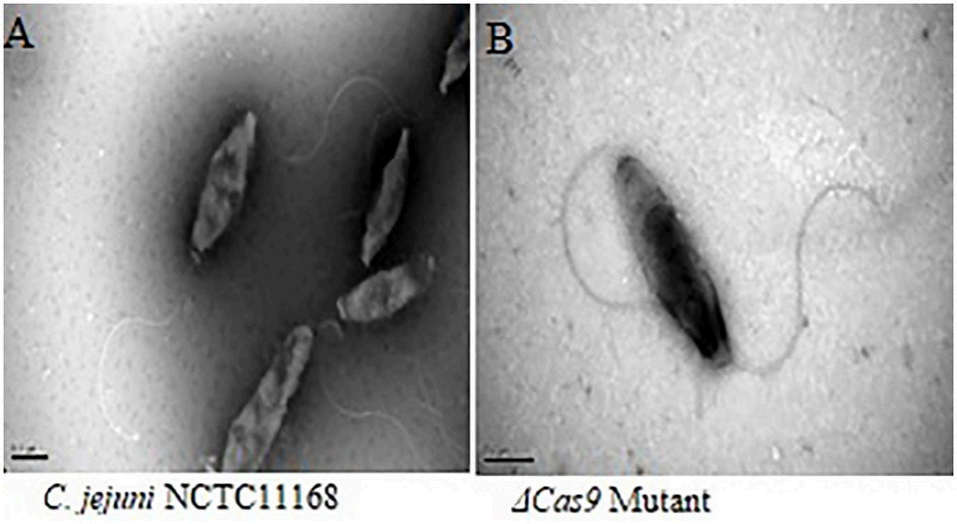
Frozen transmission electron microscopy of the *C. jejuni* NCTC 11168 and Δ*cas9* mutant strains. *C. jejuni* NCTC 11168 **(A)**, Δ*cas9* mutant **(B)**. The magnification used for TEM images in the capionis is 1μm.

*C. jejuni* NCTC11168 have polar flagella, which are necessary for motility. In present study, flagellar morphology was observed by frozen transmission electron microscopy in two strains. Apparently, no significant morphological change was found in the flagella of both *C. jejuni* NCTC 11168 and Δcas9 mutant strains (Figure 7).

### Transcriptome analysis on the basis of RNA-Seq

In order to further understand the different regulators involved in virulence, we investigated the gene expression profile of the Δ*cas9* mutant strain and compared the transcriptome profile with the *C. jejuni* NCTC11168 strain. By comparing the transcriptome profile of both strains, a total of 2.89 M reads with clean ratio 99.8% were obtained for *C. jejuni* NCTC11168. A total of 2.79 M reads with clean ratio 99.75% were obtained for the Δ*cas9* mutant strain.

Upon differential expression analysis with the *C. jejuni* NCTC11168, we found a number of important genes related to our interest were down-regulated in Δ*cas9* mutant strain (Table 1). A number of membrane encoded genes, such as *Cj1053c, Cj1203c, Cj0844c, Cj0884*, and *Cj0660c* are involved in maintaining the integrity and permeability of bacterial cell membrane. Six lipoprotein encoding genes (*Cj0983, Cj0113, Cj0950c, Cj1029c, Cj0978c*, and *Cj0842*) were also found to be down-regulated. Flagella related genes such as *Cj0547* and *Cj0440c* involved in the biosynthesis and assembly of flagella were also found to be down-regulated. *Cj0923c* (*CheR*) and *Cj0924c* (*CheB*) genes involved in chemotaxis were also found to be down-regulated. Several genes, such as *Cj0535, Cj0441*, and *Cj1299* that play a role in energy metabolism were also found to be down-regulated. Five genes (*Cj0330c, Cj0664c, Cj0471, Cj1708c*, and *Cj1694c*) encoding ribosomal proteins were also found to be down-regulated.

**Table 1:** Important down-regulated genes in Δcas9 mutant during differential expression analysis.

**Gene ID**	**Gene product**	**Fold change**	***P*-value**	***q*-value**
Cj1053c	Integral membrane protein	1.28	0.0037	0.164
Cj0844c	Integral membrane protein	1.14	0.0167	0.381
Cj0884	Integral membrane protein	1.19	0.0224	0.430
Cj0660c	Transmembrane protein	1.20	0.0485	0.623
Cj1203c	Integral membrane protein	1.29	0.0061	0.218
Cj0983	Lipoprotein	1.17	0.0497	0.623
Cj0113	Peptidoglycan associated lipoprotein	1.10	0.0313	0.496
Cj0950c	Lipoprotein	1.15	0.0266	0.460
Cj1029c	Lipoprotein (mapA)	1.16	0.0222	0.430
Cj0978c	Lipoprotein	1.37	0.0124	0.330
Cj0842	Lipoprotein	1.30	0.0060	0.218
Cj1331	Acylneuraminate cytidylyltransferase (ptmB)	1.21	0.0406	0.553
Cj0547	Flagellar protein FlaG possibly involved in flagella export	1.20	0.0117	0.327
Cj0440c	Transcriptional regulator	1.35	0.0039	0.164
Cj0535	2-oxoglutarate-acceptor oxidoreductase subunit (OorD)	1.28	0.0069	0.223
Cj0441	Acyl carrier protein carries the fatty acid chain in fatty acid biosynthesis (acpP)	1.22	0.0024	0.134
Cj1299	Acyl carrier protein (acpP2)	1.11	0.0205	0.429
Cj0330c	50S ribosomal protein L32 (rpmF)	1.13	0.0261	0.460
Cj0664c	50S ribosomal protein (rplI)	1.10	0.0209	0.429
Cj0471	50S ribosomal protein (rpmG)	1.15	0.0143	0.350
Cj1708c	30S ribosomal protein S10 NusE (rpsJ)	1.20	0.0122	0.330
Cj1694c	30S ribosomal protein (rpsN)	1.28	0.0024	0.134
Cj0923c	MCP protein methyltransferase (CheR)	1.14	0.0144	0.350
Cj0924c	MCP protein-glutamate methylesterase (cheB)	1.15	0.0189	0.406
Cj0304c	Biotin synthesis protein (bioC)	1.13	0.0267	0.460

Apart from down-regulated genes, several up-regulated genes were also observed in the present study. *Cj0551*, responsible for encoding elongation factor P, *Cj0725c* encode molybdenum cofactor biosynthesis protein, *Cj0836* encode methylated DNA protein systeine methyltransferase, *Cj0996* encode GTP cyclohydrolase II and *Cj1587c* encode putative ABC transporter proteins (Table 2). Furthermore, detail of additional DEGs were given in the S4 Table.

**Table 2:** Up-regulated genes in Δ*cas9* mutant during differential expression analysis.

**Gene ID**	**Gene product**	**Fold change**	***P*-value**	***q*-value**
Cj0551	Elongation factor P	2.09	2.67E−04	0.0195
Cj0725c	Molybdenum cofactor biosynthesis protein	2.45	3.71E−06	5.01E−04
Cj0836	Methylated DNA protein cysteine methyltransferase	2.38	4.25E−15	1.49E−12
Cj0996	GTP cyclohydrolase II	3.08	1.07E−04	0.0093
Cj1587c	Putative ABC transporter	2.70	0.039	0.544

### Verification by RT-qPCR

Seven genes (*Cj1523c, Cj0762c, Cj1222c, Cj0996, Cj0725c, Cj0454c, Cj0330c*) encoding proteins related to the virulence mechanism of *C. jejuni* NCTC1168 were selected for validation of RNA-Seq results. Among the seven selected genes only *Cj1523c* was down-regulated in the Δ*cas9* mutant strain. Other genes such as *Cj0762c, Cj1587c, Cj1222c, Cj0996, Cj0725c, Cj0454c*, and *Cj0330c* were up-regulated in the Δ*cas9* mutant strain. For these seven genes, a trend of expression level by RT-qPCR was similar to that of the RNA-Seq with a correlation coefficient of r = 0.938, which indicated a strong correlation between the two techniques (S1 Figure).

## Discussion

Over the past decade, *C. jejuni* has been reported as the leading cause of food poisoning in Europe (Bronnec et al., 2016). Due to its public health or zoonotic significance, it is important to understand the pathogenic mechanisms adopted by this pathogen, because the pathogenicity of this bacterium is largely unknown (Young et al., 2007). The CRISPR-cas system, which is an adaptive immune system of bacteria, also plays its part in controlling endogenous transcription as well as regulating bacterial pathogenicity. Similarly, *C. jejuni* has a type II CRISPR-cas system, and the functional protein of this system is *cas9*; therefore, this system has the ability to regulate bacterial pathogenesis (Shabbir et al., 2016). Therefore, in the present study we determined the pathogenesis of both *C. jejuni* NCTC1168 and its Δ*cas9* mutant strains.

*Campylobacter jejuni* NCTC 11168 has the ability to form biofilm, which is an important determinant of virulence. Biofilm develops in response to the aggregation of bacterial cells, which are surrounded by a self-producing matrix layer and adhere to the host surface. Moreover, biofilm protects the bacteria from different environmental stresses and provides resistance against the response of the host immune system (Bronnec et al., 2016). We observed in our study that biofilm formation was time dependent in *C. jejuni* NCTC11168 and this strain has a higher ability to form biofilm as compared to the Δ*cas9* mutant strain. A recent study also supports our findings that the *C. jejuni* wild strain has more capability to form biofilm as compared to mutant strains (Teh et al., 2017). This showed that the *cas9* gene might have a role in biofilm formation or enhancing virulence.

Adherence and invasion of *C. jejuni* in the host epithelial cells is considered as the main feature of its pathogenesis. There are several proteins present on the surface of bacterial cells that help the bacteria in adherence to the host cell, such as *Cj1478c* (cadF; Jin et al., 2001), and bacterial invasion into the epithelial cells is achieved by a number of secretory proteins (Konkel et al., 2004). Scientists in 2011 reported that the *C. jejuni* strain has a higher adhesion and invasion rate than its mutant strain (Almofti et al., 2011). In our *in vitro* study, we also got the same findings in *C. jejuni* NCTC11168 and its Δ*cas9* mutant strain. Based on this finding, someone can guess that the *cas9* gene might play its part in the regulation of these genes.

In light of several studies, it was observed that *C. jejuni* has the ability to multiply and survive within the macrophage cells (Day et al., 2000; Hickey et al., 2005; Iovine et al., 2008). In the present study, intracellular survivability of *C. jejuni* NCTC11168 and its Δ*cas9* mutant strain were also determined. We observed that intracellular survivability of the Δ*cas9* mutant was less than its wild strain. Our finding was in line with a previous study that also found that intracellular survivability of *C. jejuni* is greater than that of its mutant strain (Almofti et al., 2011).

Upon the transcriptome analysis of *C. jejuni* NCTC11168 and its Δ*cas9* mutant strains, we observed a number of down-regulated genes that play a role in enhancing bacterial virulence directly or indirectly. Of which the important gene is *Cj1331* (*PtmB*) involved in the glycosylation (McNally et al., 2007). Glycosylation helps in the modification of outer membrane proteins (OMPs) as well as in the assembly of flagella. Furthermore, it was also reported that *PtmB* mutagenesis does effect the motility of bacteria (Howard et al., 2009). Proteins related to outer membrane play role in maintaining the integrity and permeability of bacterial membrane (Gotoh et al., 1989). In the present study, several putative membrane associated genes (*Cj0884, Cj0884c, Cj1203c, Cj1053c, Cj0842, Cj0978c*) were found down-regulated that are additional candidates for virulence. As it was reported that membranous proteins essential for adhesion and invasion (Ye et al., 2015). Flagella formation is a key in the pathogenesis of *campylobacter* in terms of motility, biofilm formation, and adherence to and invasion of the host cell (Guerry, 2007). In the current study, we found repressed expression of genes involved in the biosynthesis and assembly of flagella such as *Cj0440c* and *Cj0547* (*FlaG*) which leads to reduced flagellar motility. Our findings are in line with those of previous study in which they reported that *Cj0440c* involved in the biosynthesis of flagella (Hao et al., 2017). According to our findings, it can be speculated that *cas9* might play a role in regulating the genes responsible for membrane integrity and adherence to the host cells as well as genes involved in the assembly and biosynthesis of flagella. This might be one reason we found less virulence in the Δ*cas9* mutant strain as compared to the wild strain.

Chemotaxis is a process in which bacteria move toward or away from chemical stimuli such as chemoeffectors or ligands that can either attractants or repellents present in the environment. By using this process, *C. jejuni* enable themselves in term of adaptation against disparate niches. In present study, we found two enzymes such as methylesterase *CheB* and methyltransferase *CheR* with repressed expression. These two enzymes involved in chemotaxis system of *Campylobacter* spp. (Chandrashekhar et al., 2017). Down-regulation of these two important enzymes of chemotaxis system suggest that *cas9* gene also play its part in the enhancement of virulence by regulating this system.

Acyl carrier protein (ACP) plays a key role in the biosynthesis of bacterial fatty acids (Martinez et al., 2010). Fatty acids play a multifaceted role in maintaining the viability and virulence of bacteria (Ma et al., 2017). In the present study, two ACP genes (*Cj0441*, and *Cj1299*) involved in fatty acid biosynthesis were found down-regulated, which explained altered pathogenic characteristics in the Δ*cas9* mutant strain compared to the wild strain. Moreover, *Cj0304c* (*bioC*) encoding biotin synthesis protein was also found to be down-regulated in present study. Biotin plays an important role in the transfer of CO_2_ in key metabolic processes such as carboxylation, decarboxylation or transcarboxylation reactions (Bi et al., 2016). *BioC* enzyme that methylates ω-carboxyl group of malonyl-ACP to form malonyl-ACP methyl ester, this molecule is accepted by the fatty acid synthesis pathway (Bi et al., 2016). Additionally, *Cj0535* (*oorD*) gene which play role in the generation of Succinyl-CoA via 2-oxoglutarate: acceptor oxidoreductase during citrate cycle (TCA cycle) was found down-regulated in current study. It was reported that *oorD* as well as *porD* (pyruvate:flavodoxin oxidoreductase) seems to be essential for bacterial survival (Su et al., 2006). Our result also supported by previous study in which they also found that *oorD* necessary for *H. pylori* survival (Su et al., 2006).

**Figure 8:**
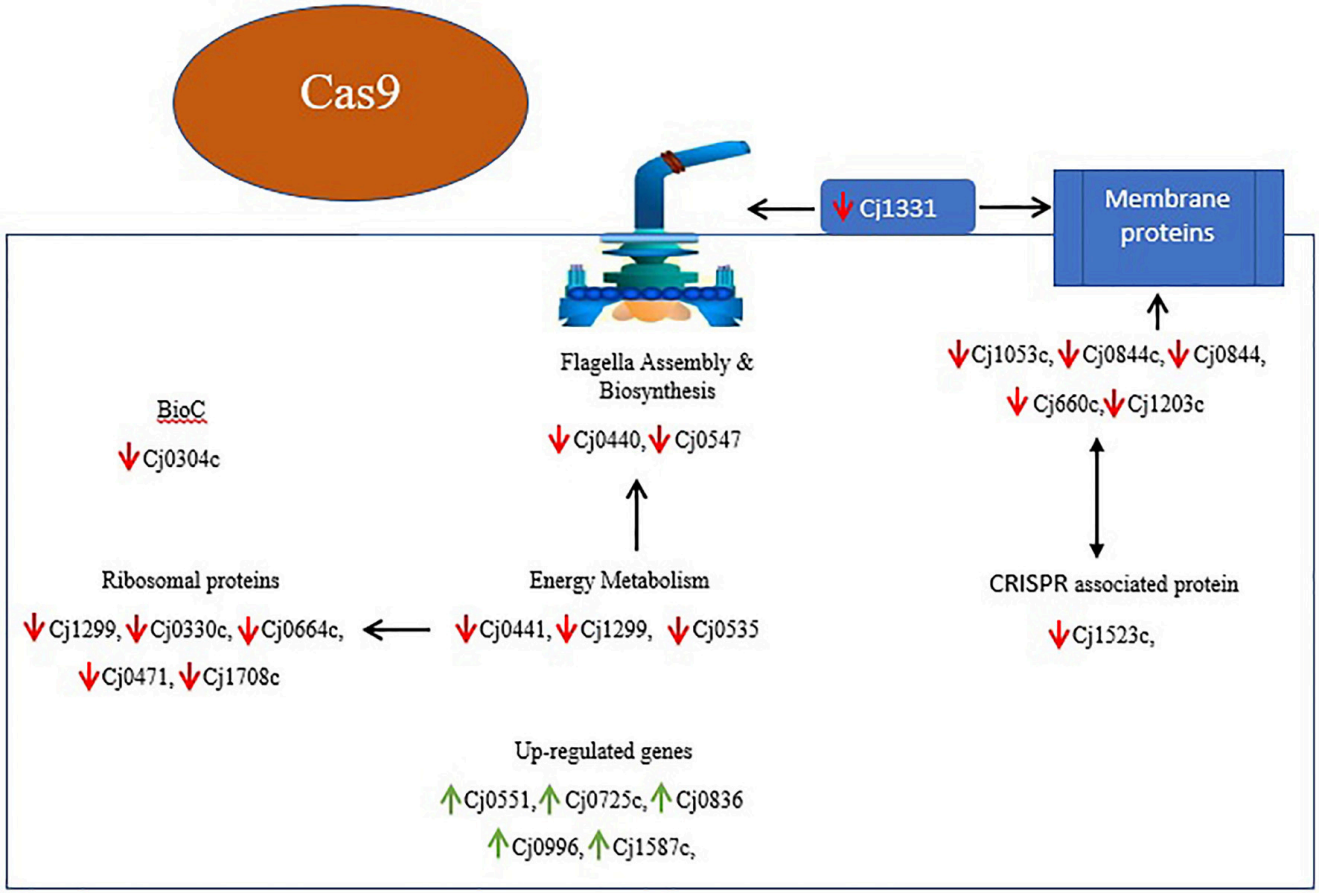
Overview of DEGs and their relationship in Δ*cas9* mutant strain on the basis of KEGG and STRING protein network analysis. Genes with red arrows indicate down-regulation, while genes with green arrows indicate up-regulation. Black double sided arrows indicate possible gene interaction.

In light of several studies, it is assumed that ribosomal proteins involved in various processes which ultimately helps in the survival of bacteria. They reported that increased expression of ribosomal proteins facilitates the multiplication and survivability of *C. jejuni* (Cecchini et al., 2007; Flint et al., 2010). However, in current study down-regulated expression of ribosomal proteins such as *Cj1694c* (*rpsN*), *Cj1708c* (*rpsJ*), *Cj0471* (*rpmG*), *Cj0664c* (*rplI*), and *Cj0330c* (*rpmF*) was observed. This suggest that *cas9* have role in the regulation of ribosomal proteins or in translation of several proteins which are essential for the survivability of bacteria (Figure 8).

## Conclusion

In conclusion, it can be speculated based on the phenotypic determination of virulence characteristics along with transcriptomic profiling that CRISPR-*cas9* is involved in the regulation of numerous virulence associated genes and enhanced the virulence of *C. jejuni*, which has zoonotic importance. Our study elucidates the genetic picture of *C. jejuni* in term of their up or down-regulation after the deletion of *cas9*. We can say in other words without any doubt that the CRISPR-*cas9* system can change the pathophysiology of *C. jejuni0*. However, there is further need to explore the potential by which *cas9* enhances virulence in *C. jejuni*.

## Author contributions

YT, HH, and ZY conceived and designed the experiments. ZX and ML performed the experiments. YT and MS analyzed the data. GC, MD, XW, ZL, HH, and ZY contributed reagents, materials, analysis tools. YT and MS wrote the paper. All authors discussed the results and commented on the manuscript.

### Conflict of interest statement

The authors declare that the research was conducted in the absence of any commercial or financial relationships that could be construed as a potential conflict of interest.
