# Supplementary material for: The Involvement of the Cas9 Gene in Virulence of Campylobacter jejuni
Source: Front Cell Infect Microbiol. 2018 Aug 20;8:285. doi: 10.3389/fcimb.2018.00285 (PMC6109747; doi:10.3389/fcimb.2018.00285)
Supplement: Supplementary file 1 [file Table_1.DOCX]

S1 Table. Primers for [homologous](javascript:void(0);) [arm](javascript:void(0);) by PCR

| Primer name | Primer sequence | Fragment length |
| --- | --- | --- |
| 3H-F1 | GCAGAACTTAAACCCTTGCAAATACG | 1272bp |
| 3H-R1 | AGTGATTTAGCCTTGCTTTG |  |
| 5H-F1 | TGCTTTTACAAGCTCTACTG | 1235bp |
| 5H-R1 | GCTGTTACAACATCGAGCTTGATAG |  |
| kan-F2 | CAAAGCAAGGCTAAATCACT CCGGAATTGCCAGCTGGGGC | 1117bp |
| kan-R2 | CAGTAGAGCTTGTAAAAGCA GTCTGACGCTCAGTGGAACG |  |
