# Supplementary material for: The Involvement of the Cas9 Gene in Virulence of Campylobacter jejuni
Source: Front Cell Infect Microbiol. 2018 Aug 20;8:285. doi: 10.3389/fcimb.2018.00285 (PMC6109747; doi:10.3389/fcimb.2018.00285)
Supplement: Supplementary file 2 [file Table_2.DOCX]

S2 Table. Primers for identification of mutant and complemented mutant

| Primer name | Primer sequence | Fragment length |
| --- | --- | --- |
| 3H-F1 | GCAGAACTTAAACCCTTGCAAATACG | 5034bp/3584bp |
| 5H-R1 | GCTGTTACAACATCGAGCTTGATAG |  |
| 16S-F | ATCTAATGGCTTAACCATTAAAC | 857bp |
| 16S-R | GGACGGTAACTAGTTTAGTATT |  |
| MapA-F | CTATTTTATTTTTGAGTGCTTGTG | 589bp |
| MapA-R | GCTTTATTTGCCATTTGTTTTATTA |  |
| kan-F2 | CAAAGCAAGGCTAAATCACT CCGGAATTGCCAGCTGGGGC | 0bp/1117bp |
| kan-R2 | CAGTAGAGCTTGTAAAAGCA GTCTGACGCTCAGTGGAACG |  |
| Cas9-F | TAAAATAAAGCGCCGCCGCAA | 3915bp |
| Cas9-R | GCAGTAAAGCCTGCCACACT |  |
| Cas9-COMP-U-F | **GCGGCCGC**ATGTTTGTAGCACTTA | 3910bp |
| Cas9-COMP-D-R | **CGCCGGCG**TCATTTTTTAAAATCTTCTC |  |
