# Supplementary material for: The Involvement of the Cas9 Gene in Virulence of Campylobacter jejuni
Source: Front Cell Infect Microbiol. 2018 Aug 20;8:285. doi: 10.3389/fcimb.2018.00285 (PMC6109747; doi:10.3389/fcimb.2018.00285)
Supplement: Supplementary file 3 [file Table_3.DOCX]

S3 Table: Primers for quantitative real-time PCR

| Primer name | Primer sequence | Fragment length |
| --- | --- | --- |
| cj0330c-F2 | GTGCAGCTAAACGCAGAACT | 92bp |
| cj0330c-R2 | ATTCGCACGGTGAGGCATTT |  |
| cj0454c-F2 | CCGCTTAGCCATTAAAGCGAC | 132bp |
| cj0454c-R2 | GAGATACCTTGCTCTCCTGCT |  |
| 16S-F | ATCTAATGGCTTAACCATTAAAC | 350bp |
| 16S-R | GGACGGTAACTAGTTTAGTATT |  |
| cj0725c-F2 | TACAGGTCCAGCACTTCGTG | 224bp |
| cj0725c-R2 | TAGCCGGAAAAACAGGCTCT |  |
| cj0996-F2 | AAATGCGACTGCGGAGAACA | 102bp |
| cj0996-R2 | AAGCCCTATACCCCTTCCTTCT |  |
| cj1222c-F2 | TTGCGGATACAGGTTGTGGA | 91bp |
| cj1222c-R2 | CCAAATCCTCCTTGATCGGT |  |
| cj1587c-F2 | TTAAGCCAAGCAAAGGCGGA | 323bp |
| cj1587c-R2 | ATCTTGATCAGCCGCCCATT |  |
| cj1523c-F2 | TTGCTAGACGCAAAGCAAGG | 223bp |
| cj1523c-R2 | TCATAGCCACGCCTTTTTGC |  |
| cj0762c-F2 | CCAGGATGGCGTTTTGGCTA | 122bp |
| cj0762c-R2 | GCAGGAATGGCTGCATGTTG |  |
