# Supplementary material for: The Involvement of the Cas9 Gene in Virulence of Campylobacter jejuni
Source: Front Cell Infect Microbiol. 2018 Aug 20;8:285. doi: 10.3389/fcimb.2018.00285 (PMC6109747; doi:10.3389/fcimb.2018.00285)
Supplement: Supplementary file 4 [file Table_4.DOCX]

**S4: Important DEGs in Δcas9 mutant strain during differential expression analysis**

| **Gene ID** | **Gene product** | **Fold change** | **P-value** | **q-value** |
| --- | --- | --- | --- | --- |
| Cj1289 | Possible periplasmic protein | 1.87 | 4.36E-07 | 6.96E-05 |
| Cj1154c | Putative cytochrome oxidase maturation protein cbb3-type | 1.85 | 1.36E-05 | 0.0015875 |
| Cj1665 | Putative lipoprotein thiredoxin | 1.64 | 3.39E-05 | 0.0037242 |
| Cj0323 | Hypothetical protein | 1.5 | 6.03E-05 | 0.0060397 |
| Cj0916c | Conserved hypothetical protein | 1.64 | 1.06E-04 | 0.0093461 |
| Cj0879c | Putative periplasmic protein | 1.55 | 2.35E-04 | 0.0179838 |
| Cj0831c | tRNA (uracil-5-)-methyltransferase | 1.63 | 9.43E-04 | 0.0571061 |
| Cj0990c | Hypothetical protein | 1.85 | 0.0016949 | 0.0992666 |
| Cj0418c | Hypothetical protein | 1.97 | 0.00472 | 0.1884769 |
| Cj0429c | Conserved hypothetical protei | 1.57 | 0.0049572 | 0.1893445 |
| Cj1309c | Hypothetical protein | 1.89 | 0.0540974 | 0.6478802 |
| Cj0989 | Hypothetical protein | 1.16 | 5.26E-10 | 1.54E-07 |
| Cj0419 | Histidine triad (HIT) family protein | 1.4 | 6.33E-04 | 0.0412012 |
| Cj0080 | Hypothetical protein | 1.25 | 0.003057 | 0.1491981 |
| Cj0376 | Hypothetical protein | 1.38 | 0.0033312 | 0.154024 |
| Cj0898 | Histidine triad (HIT) family protein | 1.22 | 0.0037466 | 0.1645674 |
| Cj0519 | Rhodanese-like domain-containing protein | 1.23 | 0.004777 | 0.188477 |
| Cj0878 | Hypothetical protein | 1.26 | 0.005957 | 0.218058 |
| Cj0717 | ArsC family protein | 1.27 | 0.006199 | 0.218186 |
| Cj1063 | Acetyltransferase | 1.29 | 0.007146 | 0.224214 |
| Cj1716c | (leuD)isopropylmalate isomerase small subunit catalyzes the isomerization between 2-isopropylmalate and 3-isopropylmalate in leucine biosynthesis | 1.35 | 0.007929 | 0.240182 |
| Cj1475c | Hypothetical protein | 1.19 | 0.008413 | 0.250529 |
| Cj0854c | Hypothetical protein | 1.17 | 0.008831 | 0.258604 |
| Cj0758 | (grpE)heat shock protein GrpE with DnaK and DnaJ acts in response to hyperosmotic and heat shock by preventing the aggregation of stress-denatured proteins | 1.18 | 0.009292 | 0.267631 |
| Cj0647 | HAD-superfamily hydrolase | 1.3 | 0.011125 | 0.315277 |
| Cj0243c | Hypothetical protein | 1.3 | 0.011987 | 0.329081 |
| Cj0639c | Adenylate kinase essential enzyme (adK) that recycles AMP in active cells | 1.14 | 0.012653 | 0.331813 |
| Cj1473c | ATP/GTP-binding protein (ctsP) | 1.22 | 0.012822 | 0.331813 |
| Cj0166 | (miaA) tRNA delta(2)-isopentenylpyrophosphate transferase IPP transferase | 1.17 | 0.012982 | 0.331813 |
| Cj0988c | Hypothetical protein | 1.19 | 0.013215 | 0.331813 |
| Cj0993c | Hypothetical protein | 1.24 | 0.014607 | 0.350152 |
| Cj1159c | Hypothetical protein | 1.26 | 0.017593 | 0.391285 |
| Cj0374 | Nucleotide-binding protein | 1.16 | 0.018957 | 0.40617 |
| Cj1021c | Hypothetical protein | 1.13 | 0.020578 | 0.429636 |
| Cj0229 | Acetyltransferase | 1.25 | 0.020754 | 0.429636 |
| Cj0900c | Hypothetical protein | 1.16 | 0.020916 | 0.429636 |
| Cj0539 | Hypothetical protein | 1.19 | 0.021572 | 0.429636 |
| Cj1361c | Hypothetical protein | 1.26 | 0.02201 | 0.429685 |
| Cj0616 | phosphate ABC transporter ATP-binding protein (pstB) | 1.15 | 0.022493 | 0.430127 |
| Cj0659c | Hypothetical protein | 1.17 | 0.022628 | 0.430127 |
| Cj0417 | Hypothetical protein | 1.12 | 0.024887 | 0.460271 |
| Cj1247c | Hypothetical protein | 1.15 | 0.024948 | 0.460271 |
| Cj0974 | Hypothetical protein | 1.11 | 0.024964 | 0.460271 |
| Cj1447c | Capsule polysaccharide export ATP-binding protein (kpsT) | 1.14 | 0.025199 | 0.460271 |
| Cj0030 | Hypothetical protein | 1.25 | 0.025487 | 0.460271 |
| Cj0792 | Hypothetical protein | 1.13 | 0.025668 | 0.460271 |
| Cj1025c | Hypothetical protein | 1.12 | 0.02577 | 0.460271 |
| Cj0012c | Non-heme iron protein (rrc) | 1.11 | 0.028241 | 0.468101 |
| Cj1488c | Cb-type cytochrome C oxidase subunit IV (ccoQ) | 1.16 | 0.029932 | 0.491503 |
| Cj0623 | Hydrogenase isoenzymes formation protein (hypB) | 1.21 | 0.029977 | 0.491503 |
| Cj1216c | Hypothetical protein | 1.21 | 0.030631 | 0.493744 |
| Cj0761 | Hypothetical protein | 1.11 | 0.032551 | 0.510638 |
| Cj0411 | ATP/GTP binding protein | 1.21 | 0.032848 | 0.510737 |
| Cj0808c | Hypothetical protein | 1.27 | 0.036318 | 0.531757 |
| Cj0899c | (thiJ) 4-methyl-5(beta-hydroxyethyl)-thiazole monophosphate synthesis protein | 1.28 | 0.037076 | 0.53837 |
| Cj0152c | Hypothetical protein | 1.21 | 0.037803 | 0.544431 |
| Cj0724 | Hypothetical protein | 1.14 | 0.038086 | 0.544431 |
| Cj0944c | Hypothetical protein | 1.2 | 0.038159 | 0.544431 |
| Cj0495 | Methyltransferase domain-containing protein | 1.18 | 0.042137 | 0.565151 |
| Cj1671c | Hypothetical protein | 1.16 | 0.043021 | 0.572636 |
| Cj0997 | Methyltransferase | 1.11 | 0.044423 | 0.586856 |
| Cj0699c | Glutamine synthetase (glnA) | 1.35 | 0.045823 | 0.600832 |
| Cj0120 | Recombination protein RecO | 1.19 | 0.047005 | 0.611767 |
