# Supplementary figures and images for: The Involvement of the Cas9 Gene in Virulence of Campylobacter jejuni
Source: Front Cell Infect Microbiol. 2018 Aug 20;8:285. doi: 10.3389/fcimb.2018.00285 (PMC6109747; doi:10.3389/fcimb.2018.00285)

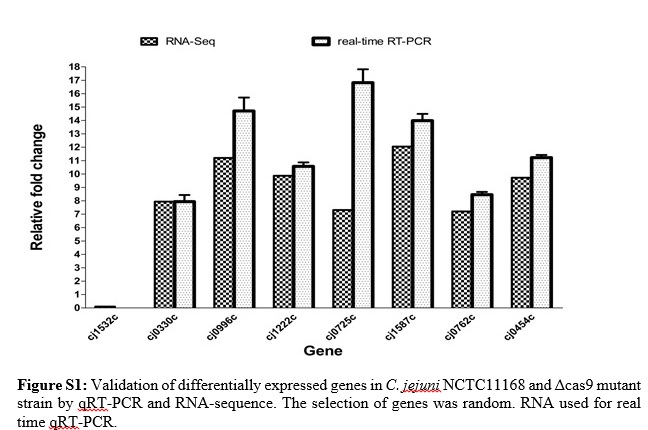

Supplement: Supplementary file 5 [file Image_1.JPEG]
